# Identifying chemogenetic interactions from CRISPR screens with drugZ

**DOI:** 10.1186/s13073-019-0665-3

**Published:** 2019-08-22

**Authors:** Medina Colic, Gang Wang, Michal Zimmermann, Keith Mascall, Megan McLaughlin, Lori Bertolet, W. Frank Lenoir, Jason Moffat, Stephane Angers, Daniel Durocher, Traver Hart

**Affiliations:** 1 Department of Bioinformatics and Computational Biology, The University of Texas MD Anderson Cancer Center, Houston, TX USA; 2 UTHealth Graduate School of Biomedical Sciences, The University of Texas MD Anderson Cancer Center, Houston, TX USA; 3 Lunenfeld-Tanenbaum Research Institute, Mount Sinai Hospital, 600 University Avenue, Toronto, ON M5G 1X5 Canada; 4 Department of Pharmaceutical Sciences, Leslie Dan Faculty of Pharmacy, University of Toronto, Toronto, ON Canada; 5 Donnelly Centre, University of Toronto, Toronto, ON Canada; 6 Department of Molecular Genetics, University of Toronto, Toronto, ON M5S 3E1 Canada; 7 Department of Biochemistry, University of Toronto, Toronto, ON Canada

**Keywords:** CRISPR screens, Chemogenetic interactions, Drug resistance, Synthetic lethality

## Abstract

**Background:**

Chemogenetic profiling enables the identification of gene mutations that enhance or suppress the activity of chemical compounds. This knowledge provides insights into drug mechanism of action, genetic vulnerabilities, and resistance mechanisms, all of which may help stratify patient populations and improve drug efficacy. CRISPR-based screening enables sensitive detection of drug-gene interactions directly in human cells, but until recently has primarily been used to screen only for resistance mechanisms.

**Results:**

We present drugZ, an algorithm for identifying both synergistic and suppressor chemogenetic interactions from CRISPR screens. DrugZ identifies synthetic lethal interactions between PARP inhibitors and both known and novel members of the DNA damage repair pathway, confirms KEAP1 loss as a resistance factor for ERK inhibitors in oncogenic KRAS backgrounds, and defines the genetic context for temozolomide activity.

**Conclusions:**

DrugZ is an open-source Python software for the analysis of genome-scale drug modifier screens. The software accurately identifies genetic perturbations that enhance or suppress drug activity. Interestingly, analysis of new and previously published data reveals tumor suppressor genes are drug-agnostic resistance genes in drug modifier screens. The software is available at github.com/hart-lab/drugz.

**Electronic supplementary material:**

The online version of this article (10.1186/s13073-019-0665-3) contains supplementary material, which is available to authorized users.

## Background

The ability to systematically interrogate multiple genetic backgrounds with chemical perturbagens is known as chemogenetic profiling. While this approach has many applications in chemical biology, it is particularly relevant to cancer therapy, where clinical compounds or chemical probes are profiled to identify mutations that inform on genetic vulnerabilities, resistance mechanisms, or targets [1]. Systematic surveys of the fitness effects of environmental perturbagens across the yeast deletion collection [2] offered insight into gene function at a large scale, while profiling of drug sensitivity in heterozygous deletion strains identified genetic backgrounds that give rise to increased drug sensitivity [3]. Now, with the advent of CRISPR technology and its adaptation to pooled library screens in mammalian cells, high-resolution chemogenetic screens can be carried out directly in human cells [4–7]. Major advantages to this approach include the ability to probe all human genes, not just orthologs of model organisms; the analysis of how drug-gene interactions vary across different tissue types, genetic backgrounds, and epigenetic states; and the identification of suppressor as well as synergistic interactions, which may preemptively indicate mechanisms of acquired resistance or pre-existing sources of resistant cells in heterogeneous tumor populations.

Design and analysis of CRISPR-mediated chemogenetic interaction screens in human cells can be problematic. Positive selection screens identifying genes conferring resistance to cellular perturbations typically have a high signal-to-noise ratio, as only mutants in resistance genes survive. This approach has been used to identify genes conferring resistance to targeted therapeutics, including BRAF and MEK inhibitors, as well as other drugs [5–13]. Conversely, negative selection CRISPR screens require growing perturbed cells over 10 or more doublings to allow sensitive detection of genes whose knockout leads to moderate fitness defects. Adding the detection of drug interactions to these experiments necessitates dosing at sub-lethal levels to balance between maintaining cell viability over a long time course and inducing drug-gene interactions beyond native drug effects [14–17].

In this study, we describe drugZ, an algorithm for the analysis of CRISPR-mediated chemogenetic interaction screens. We apply the algorithm to identify genes that drive normal cellular resistance to the PARP inhibitor olaparib in three cell lines. We demonstrate the greatly enhanced sensitivity of drugZ over contemporary algorithms [7, 18–20] by showing how it identifies more hits with higher enrichment for the expected DNA damage response pathway, and further how it identifies both synergistic and suppressor interactions. We further demonstrate the discovery of both synergistic and suppressor interactions in a single experiment with KRAS-mutant pancreatic cancer cell lines treated with an ERK inhibitor, and through reanalysis of published data. Interestingly, we observe a trend across several datasets where tumor suppressor genes score as drug suppressors, indicating a possible systematic source of false positives. We provide all software and data [21] necessary to replicate the analyses presented here; see “Availability of data and materials” below for links.

### Implementation

#### DrugZ algorithm

We calculate the log_2_ fold change of each gRNA in the pool by normalizing the total read count of each sample (to *n* = 10 million reads) at the same time point and taking the log ratio, for each replicate, of treated to control reads.

$$ {\mathrm{fc}}_r={\log}_2\left[\frac{\operatorname{norm}\left({T}_{t,r}\right)+\mathrm{pseudocount}}{\operatorname{norm}\left({C}_{t,r}\right)+\mathrm{pseudocount}}\right] $$

where:

- fc = fold change
- r = replicate indication
- T = treated sample
- *C* = control sample
- *t* = time point
- pseudocount = default value is 5

We estimate the variance of each fold change by calculating the standard deviation of fold changes with similar abundance in the control sample:

$$ \mathrm{sort}\left({\mathrm{fc}}_r\right)\ \mathrm{according}\ {C}_r\ \left(\mathrm{descending}=\mathrm{True}\right) $$

$$ \mathrm{eb}\_{\mathrm{std}}_{{\mathrm{fc}}_r}=\sqrt{\frac{1}{N}\sum \limits_i^N{\left({fc}_{r,i}-\mu \right)}^2} $$

where:

- $$ \mathrm{eb}\_{\mathrm{std}}_{{\mathrm{fc}}_r} $$ = estimated variance
- *N* = number of fold changes with similar abundance (default = 1000)
- *i* = guide
- fc_*r*, *i*_ = fold change for each guide in a replicate
- *μ* = 0

and then calculate a *Z*-score for each fold change using this estimate:

$$ {Z}_{{\mathrm{fc}}_{r,i}}=\frac{{\mathrm{fc}}_{r,i}}{\mathrm{eb}\_{\mathrm{std}}_{{\mathrm{fc}}_{r,i}}} $$

**Fig. 1:**
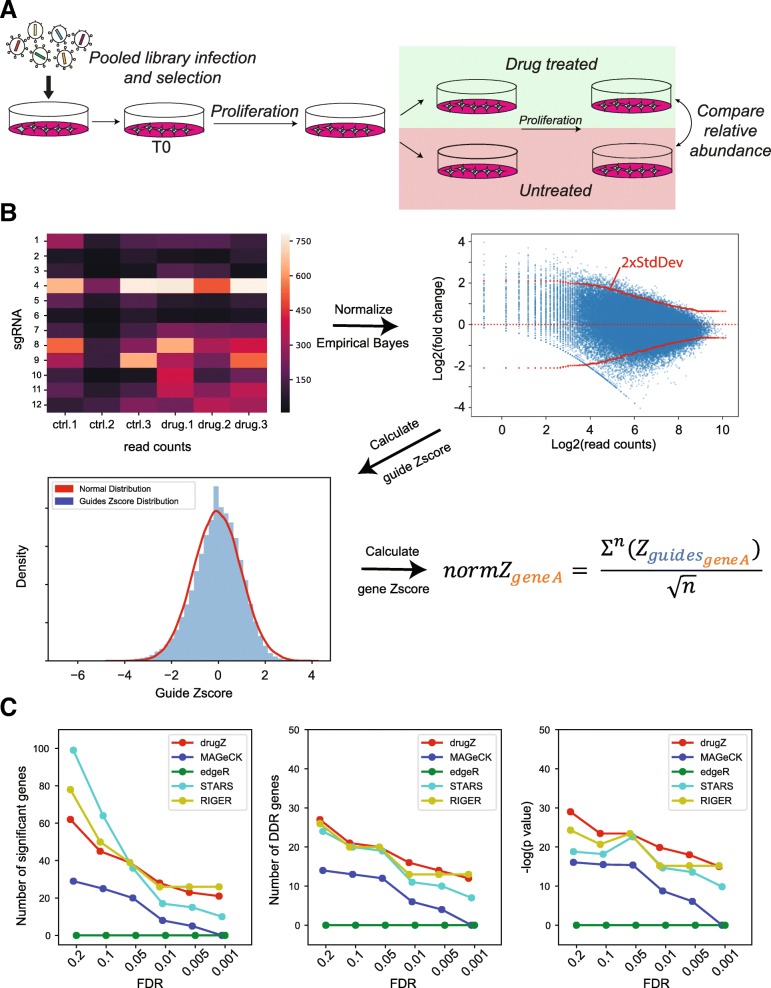
Workflow. **a** Experimental design**.** In a drug-gene interaction screen, cells are transduced with a pooled CRISPR library. Cells are split into drug-treated and untreated control samples, grown for several doublings; genomic DNA is collected; and the relative abundance of CRISPR gRNA sequences in the treated and control population is compared. **b** DrugZ processing steps include normalizing read counts, calculating fold change, estimating the standard deviation for each fold change, *Z*-score transformation, and combining guide scores into a gene score. **c–e** Comparing existing methods vs. drugZ for SUM149PT olaparib screen. DrugZ hits show strongest enrichments for DDR genes across a range of FDR thresholds. **c** Number of raw hits. **d** Number of annotated DNA damage response (DDR) genes in hits. **e** −log *P* values for DDR gene enrichment by hypergeometric test

The guide *Z*-score of all gRNA across all replicates is summed to get a gene-level sumZ score, which is then normalized (by dividing by the square root of the number of summed terms) to the final normZ (Fig. 1b):

$$ {\mathrm{normZ}}_{\mathrm{gene}A}=\frac{\sum {Z}_{{\mathrm{fc}}_{r,{i}_{\mathrm{gene}A}}}}{\sqrt{n}} $$

A *P* value is calculated from the normZ and corrected for multiple hypothesis testing using the method of Benjamini and Hochberg [22]. The open-source Python software can be downloaded from github.com/hart-lab/drugz.

#### DrugGS algorithm

After empirical Bayes variance estimation approach is applied on normalized log-fold changes to calculate a *Z*-score for each guide, we applied Gibbs sampling to generate posterior distribution of fold changes for each gene.

$$ {\displaystyle \begin{array}{c}\mathrm{P}\mathrm{osterior}\sim \mathrm{Likelihood}\ast \mathrm{Prior}\\ {}\mathrm{P}\left(\mu, \tau |\ \mathrm{data}\right)=\frac{P\left(\mathrm{data}|\ \mu, \tau \right)\ast P\left(\mu, \tau \right)\ }{P\left(\mathrm{data}\right)}\ \mathrm{posterior}\\ {}\begin{array}{c}P\left( data|\mu, \tau \right)\kern10.75em \mathrm{likelihood}\\ {}P\left(\mu, \tau \right)\kern11.25em \mathrm{prior}\ \end{array}\end{array}} $$

Each gene has a distribution composed of *Z*-scores for guides targeting that specific gene across replicates. Distribution is characterized as *ℕ*(*μ*, *τ*), where *τ* is $\frac{1}{\sigma^2}$ .

Both *μ and τ* have hyperparameters (*μ* : *μ*, *σ*^2^, *τ* : *a*, *b*) that we initialize at the very start of sampling.

*P*( *τ*| data) ~ Γ(a, b) = Gamma prior with a (shape) and b (rate) hyperparameters

*P*(*μ*| *τ*, data) ~ *ℕ*(*μ*, *σ*^2^) = Normal prior with *μ* (mean) and *σ*^2^ (variance) hyperparameters

We then update *μ and τ* with respect to their priors in every 1000 samples that we generate for each gene.

Equations to update *μ*:

$$ {\mu}_{\mathrm{update}}=\frac{\left(n\ast \overline{y}\ast \tau \right)+\left({\mu}_{\mathrm{prior}}\ast {\tau}_{\mathrm{prior}}\right)}{n\ast \tau +{\tau}_{\mathrm{prior}}} $$

$$ {\sigma}_{update}=\frac{1}{\sqrt{n\ast \tau +{\tau}_{prior}}} $$

Equations to update *τ*:

$$ {a}_{\mathrm{update}}={a}_{\mathrm{prior}}+\frac{n}{2} $$

$$ {b}_{\mathrm{update}}={b}_{\mathrm{prior}}+\sum {\left({Z}_{{\mathrm{fc}}_{r,i}}-\mu \right)}^2 $$

where:

- *n* = number of data points (guide *Z*-scores) for each gene
- $\overline{y}$ = actual mean of data points

From those 1000 newly sampled *μ* and *τ*, we then calculate the mean and standard deviation. Each gene’s *μ* posterior distribution’s mean is what was converted into *Z*-score and used to compare with the drugZ normZ values.

$$ {Z}_{\mathrm{gene}A}=\frac{\sum \limits_{k=1}^S{\mu}_k}{S} $$

where:

- *S* = number of samples (in our case 1000)
- *k* = sample

#### Drug-gene interaction screens

Olaparib screens were described in [14]. Temozolomide screens were described in [23].

#### Cell culture

hTERT RPE-1 (CRL-4000) and 293T (CRL-3216) cells were purchased from the ATCC and grown in Dulbecco’s high glucose modified Eagle’s medium (DMEM; HyClone) with 10% fetal bovine serum (FBS), 1× GlutaMAX (Gibco), 100 mM sodium pyruvate (Gibco), 1× non-essential amino acids (NEAA), 1X penicillin-streptomycin (Pen/Strep), and 5μg ml^−1^ Plasmocure. Incubator conditions were kept at 37 °C with 5% CO_2_.

#### Lentivirus production

For production of the TKOV3 lentivirus, 9.0 × 10^6^ 293T cells were transfected with psPAX2 (lentiviral packaging; Addgene #12260), pMD2.G (VSV-G envelope; Addgene #12259), and TKOV3 (Toronto KnockOut CRISPR Library; Addgene #90294) using X-tremeGENE 9 DNA transfection reagent (Sigma-Aldrich) in medium with lowered antibiotic concentration (0.1× Pen/Strep). The medium was replaced with viral harvest medium (DMEM + 1.1% BSA + 1× Pen/Strep) 18 h post-transfection. Virus-containing supernatant was collected ~ 24–48 h post-transfection, and fresh viral harvest medium was added to transfected plates. Virus-containing supernatant was collected again ~ 24 later. The virus-containing supernatant was centrifuged to remove cell debris and stored at -80 °C.

#### CRISPR screening

For transduction of the hTERT RPE-1 cells, the TKOv3 virus was added with 8μg/ml Polybrene. For selection of the transduced cells, puromycin was introduced at a concentration of 20 μg/ml at 24 h post-infection (the hTERT cassette used to immortalize RPE1 cells contains a puromycin resistance marker, necessitating extreme puromycin concentrations for selection). Puromycin selection continued for 72 h post-transduction and completed upon the selection against the hTERT RPE-1 parental line as a control. Completion of selection was considered the initial time point (*T*_0_). The TKOv3-transduced cells were split into technical replicates. To ensure proper coverage, 15 × 10^6^ cells across 11 × 15 cm dishes were used for infection with the TKOv3 virus per replicate. The chemotherapeutic drugs gemcitabine (2 nM) and vincristine (0.4 nM) were added to separate replicates, with one set of replicates receiving no drug treatment. Both drug-treated and untreated replicates were not allowed to reach confluence in the 15 cm dishes. Cells were lifted, counted, and re-plated at the coverage stated above, and the excess cell pellets were frozen at − 20 °C as a time point. Once 8 doublings were reached from *T*_0_, the screens were terminated and pellets frozen at − 20 °C. Coverage of screens was kept at 200 cells per gRNA.

The QIAamp Blood Maxi Kit (Qiagen) was used to isolate the genomic DNA (gDNA) from the frozen cell pellets. Guide sequences were enriched using PCR with HiFi HotStart ReadyMix (Kapa Biosystems) and primers targeting the guide region in the genomic DNA. A second round of PCR was performed with i5 and i7 primers to give each condition and replicate a unique multiplexing barcode. The final PCR products were purified using the E-Gel System (Invitrogen), normalized, and sequenced on the NextSeq500 system to determine the representation of guides under each treated and non-treated condition.

## Results and discussion

We created the drugZ algorithm to fill a need for a method to identify chemogenetic interactions in CRISPR knockout screens. In a pooled library CRISPR screen, the relative starting abundance of each gRNA in the pool is usually sampled immediately after infection and selection. To identify genes whose knockout results in a fitness defect (“essential genes”), the cells are grown for several doublings and the relative abundance of gRNA is again sampled by deep sequencing of a PCR product amplified from genomic DNA template. The relative frequency of each gRNA is compared to starting gRNA abundance, and genes whose targeting gRNA show consistent dropout are considered essential genes.

In a chemogenetic interaction screen, the readout is different: the relative abundance of gRNA in a treated population is compared to the relative abundance of an untreated population at a matched time point (Fig. 1a). In this context, an experimental design with paired samples should be particularly powerful, as it removes a major source of variability across replicates.

To benchmark the method, we evaluated screens to identify modifiers of the response to the PARP inhibitor olaparib in three cell lines, RPE1-hTERT, HeLa, and SUM149PT [14]. The screens were performed using the TKOv1 library of 90k gRNA targeting 17,000 genes and are described in detail in [24]. After infection and selection, each cell line was split into 3 replicates, passaged at least once, and each replicate was further split into control and olaparib-treated populations (Fig. 1a).

The drugZ algorithm calculates a fold change for each gRNA in an experimental condition relative to an untreated control. A *Z*-score for each fold change is calculated using an empirical Bayes estimate of the standard deviation, by “borrowing” information from gRNA observed at a similar frequency (read count) in the control cells. Guide-level gene scores are combined into a normalized gene-level *Z*-scores called normZ, from which *P* values are estimated from a normal distribution (Fig. 1b). We used drugZ to calculate normZ scores, *P* values, and false discovery rates in SUM149PT breast cancer cells, which carry *BRCA1* and *TP53* mutations, +/− olaparib treatment [14]. We also analyzed the same data with four contemporary methods, STARS [7], MAGeCK [18], edgeR [19], and RIGER [20]. We noted that drugZ produced a moderate number of overall hits, relative to other methods, as FDR thresholds were relaxed (Fig. 1c). We evaluated the quality of the hits by measuring their functional coherence. The PARP inhibitor olaparib was developed specifically to exploit the observed synthetic lethal relationship between *PARP1* and the *BRCA1*/*BRCA2* genes [25, 26]. Subsequent studies have shown it to be effective against a general deficiency in homologous recombination repair, known as HRD [27]. We therefore calculated the enrichment of each hit set for genes in the DNA damage response (DDR) pathway as annotated in the Reactome database [28] and found that drugZ hits show strong enrichment for DDR genes across a range of FDR thresholds (Fig. 1d, e), while the other methods show consistently lower enrichment. We observed similar trends in an olaparib screen in HeLa cells (Additional file 1: Figure S1A) but less overall effect in hTERT-immortalized RPE1 wildtype epithelial cells (Additional file 1: Figure S1B). The combination of larger sets of hits and greater enrichment for expected results indicates that drugZ accurately and sensitively identifies chemogenetic interactions.

The drugZ algorithm can also be used to identify suppressor interactions, that is, genes whose perturbation reduces drug efficacy. While *BRCA1* mutation is synthetic lethal with *PARP1*, subsequent mutation of *TP53BP1* is associated with acquired resistance to the PARP inhibitor [29]. Drug-gene interactions resulting in positive *Z*-scores reflect such suppressor interactions. Indeed, *TP53BP1* is the 8th-ranked suppressor interaction in *BRCA1*-deficient SUM149PT cells, with a normZ score of 3.05. Similarly, newly described resistance gene *C20orf196*, now called *SHLD1* [30–33], is the top-ranked suppressor.

### Robustness to parameter choice and experimental design

To evaluate the robustness of the drugZ approach, we conducted sensitivity analysis using data from the SUM149PT olaparib screen. The algorithm relies on two major tunable parameters, window size for empirical Bayes variance estimation and a monotone filter for the variance estimator (to ensure non-decreasing variance as read count decreases). The window size represents the number of neighboring gRNA, ranked by read count, to use to evaluate gRNA fold change variance. To evaluate the effect of varying window size, we ran the drugZ pipeline with window sizes in five increments from 100 to 1000; neither the number of hits, number of DDR-annotated hits, nor enrichment *P* value was affected by changing window size (Additional file 1: Figure S2a). We performed a similar analysis with and without enforcing the monotone filter and discovered marginally improved performance in the SUM149PT olaparib screen without enforcing monotonicity (Additional file 1: Figure S2b), but no such effect in Hela (T15) olaparib screen (Additional file 1: Figure S2c). We therefore left the filter in place.

We also tested the drugZ pipeline against a more statistically thorough, but computationally demanding, approach. After using the same empirical Bayes approach to calculate a *Z*-score for each guide, we applied Gibbs sampling to estimate the posterior distribution of fold changes for each gene (Additional file 1: Figure S3A). This method, which we termed drugGS, yielded results that are virtually identical to drugZ (Pearson correlation coefficient = 0.99; Additional file 1: Figure S3B) at ~ 50× the computational cost (Additional file 1: Figure S3C). DrugGS is also available on github at https://github.com/hart-lab/druggs.

### Experimental design considerations

**Fig. 2:**
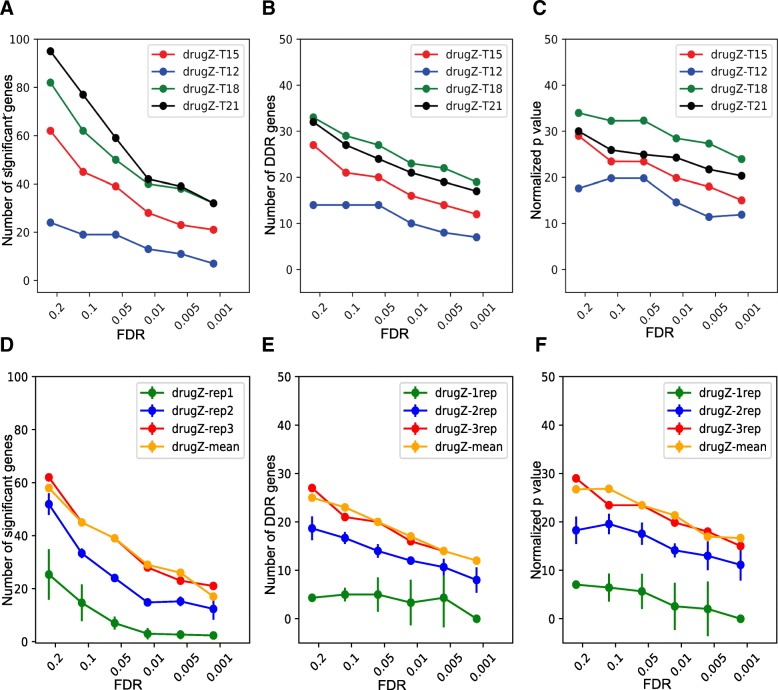
Experimental design effects. **a**–**c** DrugZ performance across different time points for SUM149PT olaparib screen. **a** Number of raw hits. **b** Number of annotated DNA damage response (DDR) genes in hits. **c** −log *P* values for DDR gene enrichment. **d–f** DrugZ performance based on varying number of replicates. **d** Number of raw hits. **e** Number of annotated DNA damage response (DDR) genes in hits. **f** −log *P* values for DDR gene enrichment. Rep1, 2, 3: all combinations of one, two, or three replicates, ± s.d. Mean: comparing mean of drug-treated samples to the mean of control samples (unpaired approach)

Highly effective CRISPR knockout screens are done with a variety of experimental designs, with varying numbers of replicates, degree of library coverage, determination of endpoint, and whether intermediate time points are included [5–7, 24, 34–40]. The olaparib drug-gene interaction screens described here were performed in triplicate in 15-cm plates and passaged every 3 days, with drug added at day 6 and samples collected for sequencing at each passage starting at day 12 [14]. Using the optimized drugZ pipeline, we evaluated each time point in the SUM149PT screens. The screen’s ability to resolve specific DNA damage response genes increased steadily from day 12 to day 18 (Fig. 2a–c), highlighting the importance of low-dose drug treatment (e.g., LD20). The extended timeframe for the experiment allows greater resolution of negative selection hits as they disappear from the population over several doublings.

Nevertheless, the screens are still quite noisy, necessitating several replicates for accurate assessment of drug-gene interactions. The experimental design of these screens involved control and drug-treated samples for each replicate, facilitating a paired-sample analysis across the three replicates (Additional file 1: Figure S4A). In contrast, an unpaired design (Additional file 1: Figure S4A) requires comparing the means (or other aggregate metric) of the treated and untreated arms. In our experience, a paired-sample experimental design typically results in within-replicate samples clustering together (Additional file 1: Figure S4B), suggesting a paired-sample analysis would be more sensitive. Paired-sample analysis of three replicates in the olaparib screen clearly outperforms one- or two-replicate designs (Fig. 2b). Surprisingly, however, the paired-sample approach does not appear to offer significant benefits over an unpaired approach: when taking the mean fold change across experimental samples and comparing it to the mean fold change across control samples (Additional file 1: Figure S4A), the results are nearly identical to analysis of three paired samples (Fig. 2d–f). Indeed, treating samples as paired or unpaired produced highly correlated results (rho> = 0.96) in all three olaparib screens (Additional file 1: Figure S4c-e), and the functional enrichment analysis in SUM149PT cells showed virtually no difference when performing paired-sample or unpaired-sample analysis (Additional file 1: Figure S4f-h).

### A general-use algorithm for drug-gene interactions

**Fig. 3:**
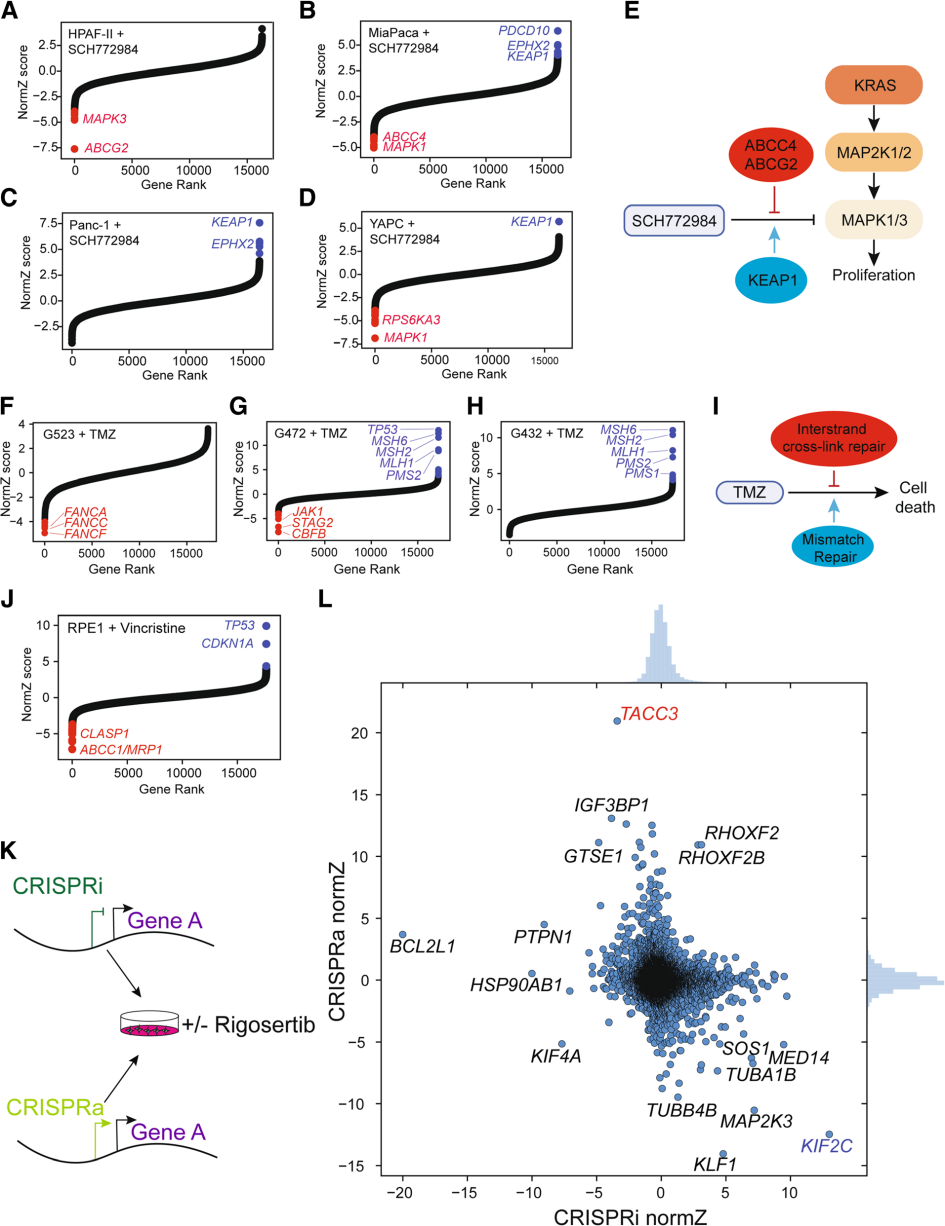
DrugZ effectiveness across diverse screens. **a**–**d** DrugZ-calculated normZ score is plotted vs. gene rank for SCH772984 screen in four KRAS pancreatic cancer cells cell lines. Synergistic/synthetic lethal (red) and suppressor/resistance (blue) interactions at FDR < 0.1. **e** Network view of ERK inhibitor screens. Red, synthetic lethal interactions. Blue, suppressor interactions. **f**–**h** Glioblastoma cell lines screened for chemogenetic interactions with temozolomide (TMZ), as described in [23]. **i** Pathway-level summary of modifiers of TMZ activity in glioblastoma cells. **j** hTERT-RPE1 cells screened for modifiers of vincristine. **k** Experimental design of CRISPRi/CRISPRa screens for modifiers of rigosertib, as described in [41]. **l** DrugZ results of the combined rigosertib screens. Red/blue hits are characterized in [41]

To ensure that the drugZ algorithm is not overspecialized for the strong chemogenetic profile of PARP inhibitors, we applied it to a separate set of drug interaction screens in pancreatic cancer cell lines using the ERK1/2 inhibitor SCH772984. Oncogenic mutations in *KRAS* drive constitutive signaling in the MAP kinase pathway and are associated with proliferation and survival signals. Consistent with current models of *RAS* pathway activation, knockout of inhibitor target *MAPK1* has strong synthetic sick/lethal or negative interactions with ERK inhibitor in two of the cell lines, MiaPaca and YAPC (FDR < 0.1; Fig. 3a–d). In the third cell line, HPAF-II, the top synthetic interactors were drug transporter *ABCG2* and *MAPK3*. Activity of this drug resistance gene may account for this cell line’s resistance to ERK inhibition and the lack of other synthetic effectors in this screen. Drug transporter *ABCC4* is synthetic lethal in MiaPaca cells, indicating multiple routes of drug resistance for this molecule. Ubiquitin ligase adapter *KEAP1* is among the top suppressors of ERK inhibitor activity in three cell lines (Fig. 3a–d). *KEAP1* loss of function was identified as a modulator of MAP kinase pathway inhibitors in a panel of positive selection screens in multiple cell lines [11], suggesting a context-dependent model for predicting ERK inhibitor activity (Fig. 3e). Notably, the ERK inhibitor screens yielded a small number of discrete synthetic and suppressor hits, in contrast with the PARP inhibitor screens, which showed broad interaction across the HR pathway, confirming the general applicability of drugZ in detecting drug-gene interactions.

We additionally reanalyzed data from a set of temozolomide (TMZ) drug modifier screens in patient-derived glioblastoma cell lines [23]. The screens clearly indicated synthetic lethality with the Fanconi anemia complex (Fig. 3f) and suppressor activity from the mismatch repair pathway (Fig. 3g, h). Together, these results recapitulate the biological drivers of temozolomide: mismatch repair is required for temozolomide cytotoxicity [42], while the Fanconi anemia pathway plays a major role in the repair of TMZ-induced damage [22, 43, 44] (Fig. 3i). We further conducted an independent screen of hTERT-immortalized RPE1 epithelial cells to determine genetic modifiers of the microtubule stabilizing agent vincristine. Drug transporter *ABCC1* (encoding multidrug resistance protein-1*,* or MRP1), a known marker for clinical resistance to vincristine [45, 46], is the top synthetic hit in our screen (Fig. 3j).

Finally, we reprocessed data from complementary CRISPRi/CRISPRa screens for modifiers of rigosertib activity [41] (Fig. 3k). As transcriptional activation and repression are expected to show opposite effects in a phenotypic screen, we plotted the drugZ results for the CRISPRi screen and the CRISPRa screen together (Fig. 3l). The microtubule stabilizing activity of *TACC3* and destabilizing activity of *KIF2C*, characterized extensively in [41], are both recovered by drugZ, along with tubulins *TUBA1B* and *TUBB4* (Fig. 3l), consistent with rigosertib’s activity as a microtubule destabilizing agent. Importantly, these results confirm the applicability of drugZ beyond CRISPR knockout screens.

**Fig. 4:**
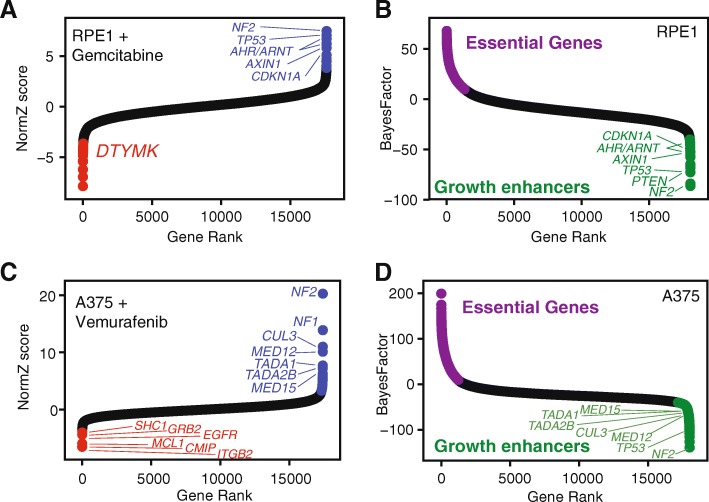
Tumor suppressor genes are frequent drug suppressor hits. **a** normZ plot hTERT-RPE1 screen for modifiers of gemcitabine activity, colored as in Fig. 3. **b** Gene essentiality of untreated hTERT-RPE1 cells. Purple, essential genes. Green, genes whose knockout imparts a fitness advantage. **c** normZ plot of A375 melanoma cell line screen for vemurafenib modifiers; data from [5]. **d** Gene essentiality scores for A375; data from [48]

We noted that a small number of genes were unexpected repeat hits across several screens using different drug or small molecule perturbagens with disparate mechanisms of action. We screened hTERT-RPE1 cells with gemcitabine, a pyrimidine nucleoside analog, and analysis with drugZ reveals a synthetic lethal interaction with deoxythymidylate kinase *DTYMK*. *DTYMK* phosphorylates dTMP to dTDP, a key step in the synthesis-by-salvage pathway of dTTP [47] (Fig. 4a). However, suppressors of gemcitabine activity included *NF2*, *TP53*, *AXIN1*, and other known tumor suppressor genes (Fig. 4a) with no known role in nucleotide metabolism. This immortalized epithelial cell line carries wildtype alleles of these tumor suppressors, and their knockout in a CRISPR screen results in cell proliferation more rapid than wildtype cells. This is reflected in the essentiality profiles, as calculated by BAGEL [49]: essential genes have positive Bayes Factors, but tumor suppressors show extreme negative scores (Fig. 4b).

We hypothesized that such tumor suppressors might be systematic, nonspecific hits in drug-gene interaction screens. We re-analyzed other screens to understand this behavior across different cell backgrounds. The landmark CRISPR screen paper from Shalem et al. [5] includes a screen in *BRAF*-mutated A375 melanoma cells for resistance to vemurafenib and describes the discovery of *NF2* as a novel suppressor of vemurafenib activity. DrugZ analysis confirms *NF2* as a strong hit in the screen, along with *NF1* and several members of the mediator complex (Fig. 4c). Complementary analysis of the gene essentiality profile for A375 derived from Behan et al. [48]—the latest screens from the DepMap project are substantially superior to the first-generation screen performed in Shalem et al., as shown by precision-recall analysis (Additional file 1: Figure S5)—shows that *NF2* is the top ranked tumor suppressor in the screen, and furthermore, virtually every other vemurafenib suppressor hit shows enhanced cell fitness when knocked out (Fig. 4d). Interestingly, we detect *MCL1* and *EGFR*, as well as *EGFR* signal transduction components *SHC1* and *GRB2*, as synthetic lethal with vemurafenib in this screen. Neither hit is reported in the original study, but both *MCL1* [50] and *EGFR* [51, 52] have been characterized as routes of adaptive resistance to *BRAF* inhibition in melanoma. These findings support the overall quality of the drug-gene interaction screen and our analysis of the data. We further note that *TP53* and *CDKN1A* (p21) are the top suppressors in the RPE1 vincristine screen (Fig. 3j) and that *TP53* is the top suppressor in the G472 temozolomide screen (Fig. 3g). G472 cells carry a wildtype p53 gene [23]. Collectively these results indicate that genes whose knockout imparts a growth advantage on cells are recurrent hits in drug-gene interaction screens, suggesting a drug-agnostic phenomenon rather than drug-specific resistance mechanisms.

## Conclusions

Identifying the genetic drivers of drug effectiveness and resistance is critical to realize the promise of personalized medicine. Chemogenetic interaction screens in mammalian cells using CRISPR knockout libraries have so far been primarily used in a positive selection format to identify the genes, pathways, and mechanisms of acquired resistance to chemotherapeutic drugs. However, negative selection screens to identify the underlying architecture of drug-gene interactions have been difficult to carry out and to analyze in part due to the lack of robust analytical tools.

We describe the drugZ algorithm, which calculates a gene-level *Z*-score for pooled library CRISPR drug-gene interaction screens. By taking into account the moderate single mutant fitness defects associated with many genes involved in drug-gene interactions, the drugZ algorithm offers significantly improved sensitivity over contemporary analysis platforms. The algorithm was developed to exploit the additional resolving power we expected to gain from a paired-sample experimental design, but surprisingly this has virtually no effect on our results. We demonstrate the validity of our hits by showing the strong enrichment for genes involved in the DNA damage response in a screen for interactions with the PARP inhibitor olaparib and the precise detection of MAPK pathway effectors in an ERK inhibitor screen. We further show that both synergistic and suppressor interactions can be identified in the same screen, as the previously identified PARP resistance gene *TP53BP1* and newly characterized *SHLD1* (formerly *C20orf196*) are top-ranked suppressors of olaparib activity in *BRCA1*-mutant SUM149PT screens. Moreover, both synthetic targets *MAPK1/3* and suppressor gene *KEAP1* are identified in ERK inhibition screens. *KEAP1* deletion or mutation is frequently found in KRAS-driven lung adenocarcinomas and may present an obstacle to ERK inhibitor therapy in these tumors.

Experimental design plays a critical role in the ability to accurately identify drug-gene interactions. Negative selection screens for synthetic lethal interactions require that cells be carried long enough for dropouts—typically growth defects rather than full synthetic lethals—to rise to statistical significance. Our results, concordant with known highly drug-specific differences in effect timing, suggest that there is value in collecting multiple time points to ensure that drug activity and genetic interaction are detectable and that traditional dose-response curves must be calculated over a time course relevant to the screen (e.g., at least two passages or several doublings).

Copy number amplifications have been widely shown to cause locus-specific, but not gene-specific, toxicity in CRISPR knockout experiments. This phenomenon can lead to false positives in screens for knockout fitness defects. However, drug-gene interaction screens measure whether, in the CRISPRko case, a double-strand break at a specific locus amplifies or suppresses the activity of a small molecule or other perturbagen. Amplification-specific artifacts should, in principle, show no difference between treated and control samples and should therefore not be a significant source of false positives. However, gRNA targeting amplified loci may rapidly drop out of a population of cells under library-induced selection; the absence of these loci at experimental end points (as measured by gRNA read counts) could feasibly mask the detection of drug-gene interactions, resulting in false negatives.

Despite these technical idiosyncrasies, chemogenetic interaction screens extend the utility of CRISPR genome-scale perturbation screens by enabling the systematic survey of the landscape of drug-gene interactions across cancer-relevant genetic backgrounds. Understanding this variation may lead to more precise therapies for patients as well as the development of synergistic drug combinations for genotype-specific treatments.

### Availability and requirements

Project name: drugz

Project home page: https://github.com/hart-lab/drugz

Operating system: platform independent

Programming language: Python

Other requirements: Python v3.7 or higher; modules numpy, scipy, pandas.

License: MIT

No restriction for non-academic use

## Additional file

Additional file 1: **Figure S1.** DrugZ vs. other methods with olaparib screens in HeLa (A) and RPE1 (B) cells. Figure S2. DrugZ tunable parameters. Figure S3. DrugZ vs. DrugGS. Figure S4. Paired vs. non-paired approaches in three olaparib screens. Figure S5. Recall-precision plot of gene essentiality screens in A375 cells. (PDF 1842 kb)

## Data Availability

All software described in this manuscript, as well as all data files used for analysis, are available (under the MIT license) at the Hart Lab github site and figshare: https://github.com/hart-lab/drugz https://github.com/hart-lab/druggs https://figshare.com/projects/DrugZ_software_from_the_Hart_Lab/65582
