# Supplementary material for: Identifying chemogenetic interactions from CRISPR screens with drugZ
Source: Genome Med. 2019 Aug 22;11:52. doi: 10.1186/s13073-019-0665-3 (PMC6706933; doi:10.1186/s13073-019-0665-3)
Supplement: Supplementary file 1 — Figure S1. DrugZ vs. other methods with olaparib screens in HeLa (A) and RPE1 (B) cells. Figure S2. DrugZ tunable parameters. Figure S3. DrugZ vs. DrugGS. Figure S4. Paired vs. non-paired approaches in three olaparib screens. Figure S5. Recall-precision plot of gene essentiality screens in A375 cells. (PDF 1842 kb) [file 13073_2019_665_MOESM1_ESM.pdf]

Supplementary Figures

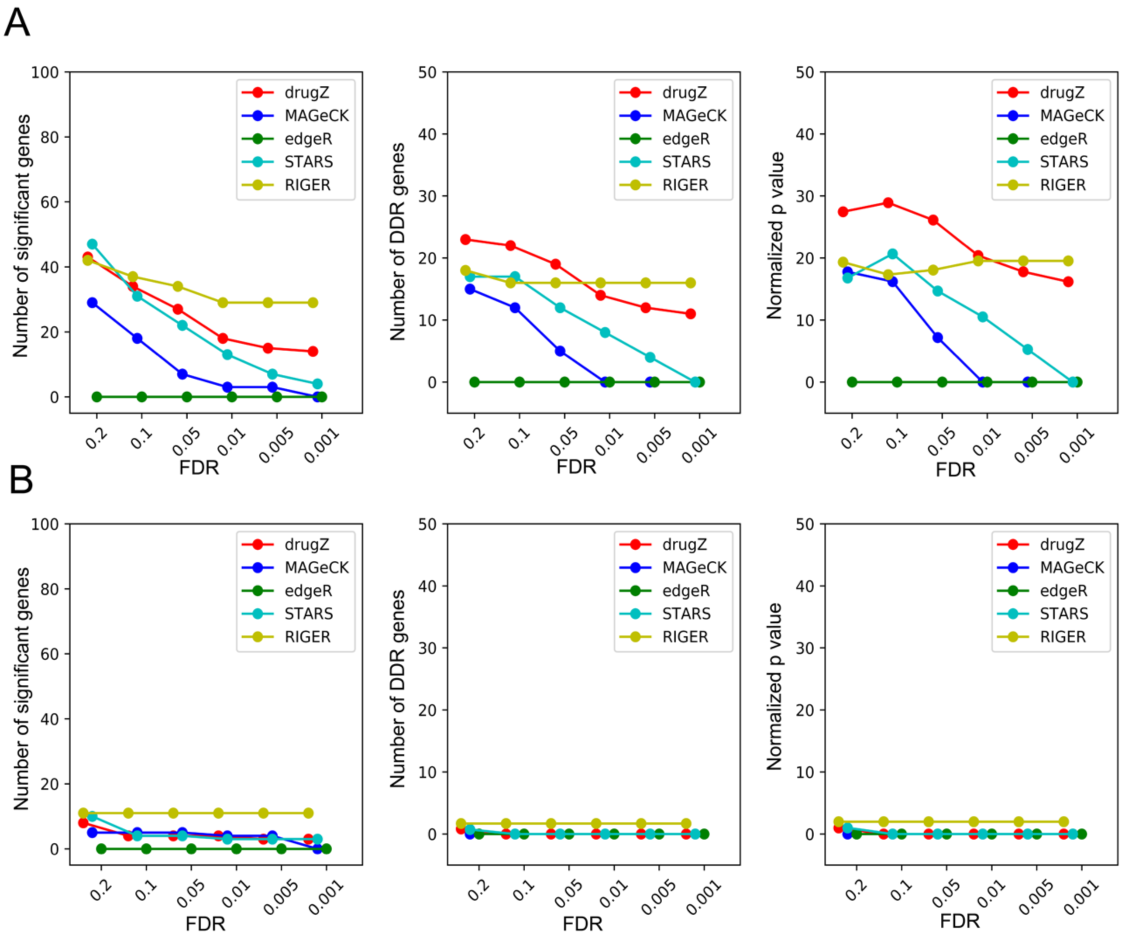

Figure S1. DrugZ vs. other methods with olaparib screens in HeLa (A) and RPE1 (B) cells. Methods are colored as in Fig.1C. DrugZ hits show strongest enrichment for DDR genes across a range of FDR thresholds in these two screens as well but less overall effect in RPE1 cells. Data from (14) **(A)** Left, number of raw hits. Center, number of annotated DNA Damage Response (DDR) genes in hits. Right, log P-values for DDR gene enrichment. **(B)** Same panels as in (A), for RPE1 screen.

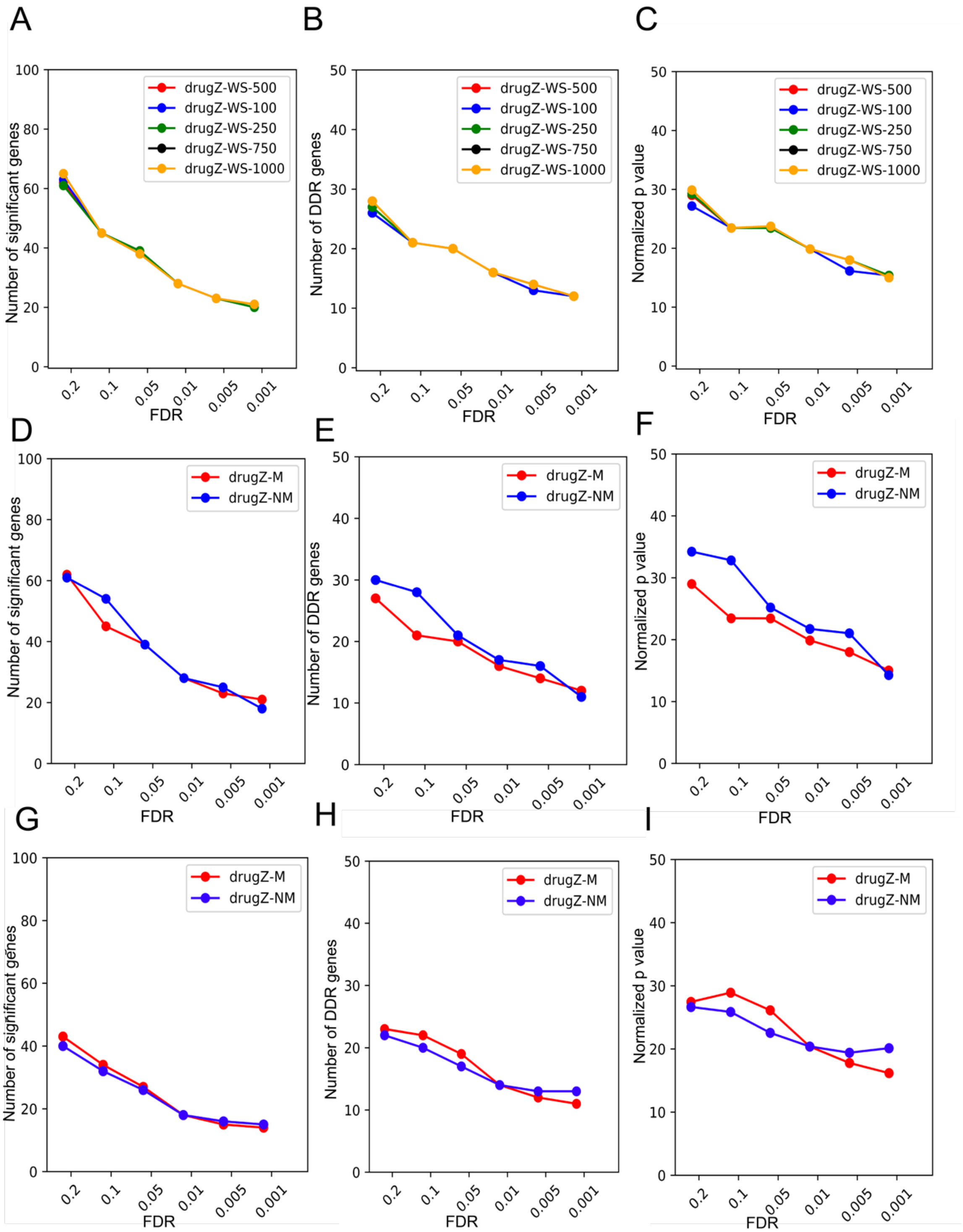

Figure S2. DrugZ tunable parameters.

(A) DrugZ performance across different window sizes for Empirical Bayes estimation of variance of guide-level fold changes. Left, number of raw hits. Center, number of annotated DNA Damage Response (DDR) genes in hits. Right, log P-values for DDR gene enrichment. **(B)** DrugZ performance with correction that ensures monotonicity in the variance (M, red) vs. drugZ performance with no correction that ensures monotonicity in the variance (NM, blue) in SUM149PT olaparib screen (panels same as in (A)). **(C)** DrugZ performance with correction that ensures monotonicity in the variance (red) vs. drugZ performance with no correction that ensures monotonicity in the variance (blue) in HeLa olaparib screen (panels same as in (A) and (B)).

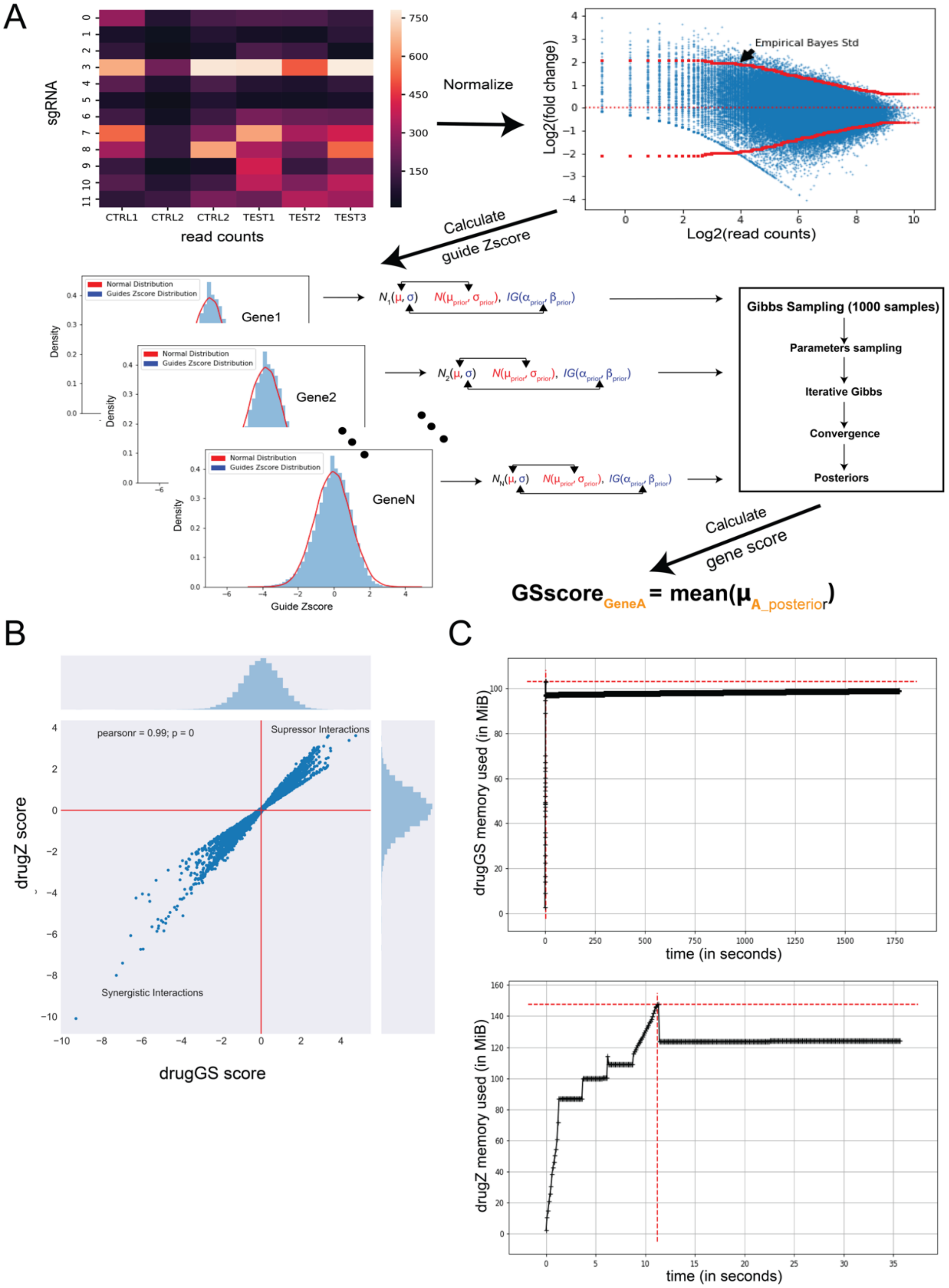

Figure S3. DrugZ vs. DrugGS

(A) DrugGS Computational Pipeline. DrugGS preprocessing steps are same as in the DrugZ for generating guide-level Z-scores. After guide level Z-scores are obtained, they are used as a prior distribution to generate gene-level scores using Gibbs sampling. The mean of generated sample of means is considered as new gene score. **(B)** Comparison between drugGS (x-axis) and drugZ (y-axis) gene scores shows high concordance between the two methods (Pearson correlation coefficient = 0.99). **(C)** Comparison between drugGS (top) and drugZ (bottom) time and memory performance. DrugZ drastically outperforms drugGS in terms of time and memory used.

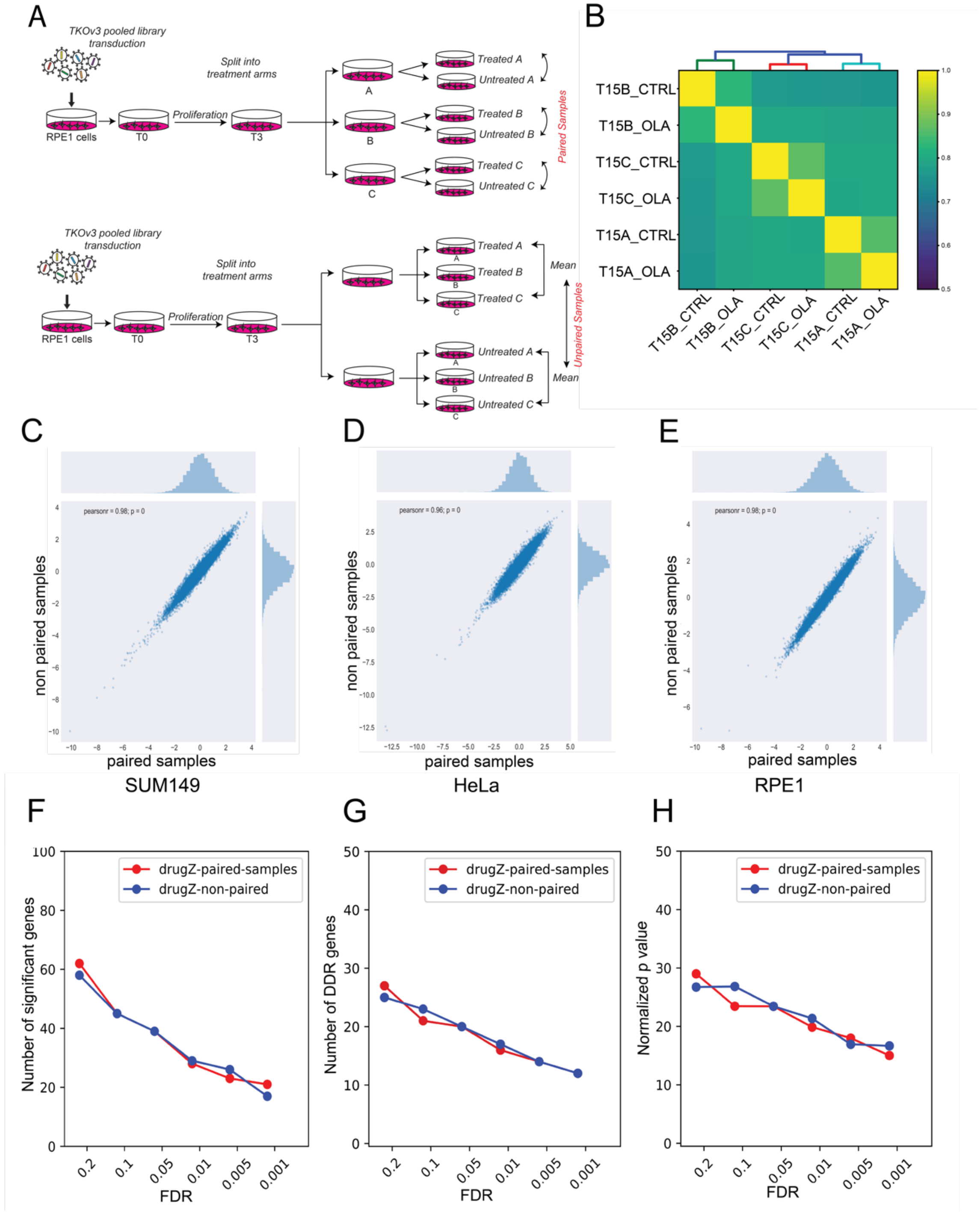

Figure S4. Paired vs. non-paired approaches in three olaparib screens.

(A) Experimental designs describing paired (top) and unpaired (bottom) experimental design and analysis strategy for chemogenetic interaction screens. (B) Clustering of gRNA-level fold changes across replicates shows that within-replicate samples correlate more closely than samples with similar treatment. (C) Correlation between paired samples (control A – treated A, control B – treated B, etc.) vs. non-paired (mean (control A, B, C) – mean (drug A, B, C.)) for SUM149PT olaparib screen (rho = 0.98) (D) Same as in (A) for HeLa olaparib screen (rho = 0.96) (E) Same as in (A) for RPE1 olaparib screen (rho = 0.98) (F-H) Comparison between paired and non-paired approaches across number of significant genes, DDR genes and normalized p-values in SUM149PT olaparib screen.

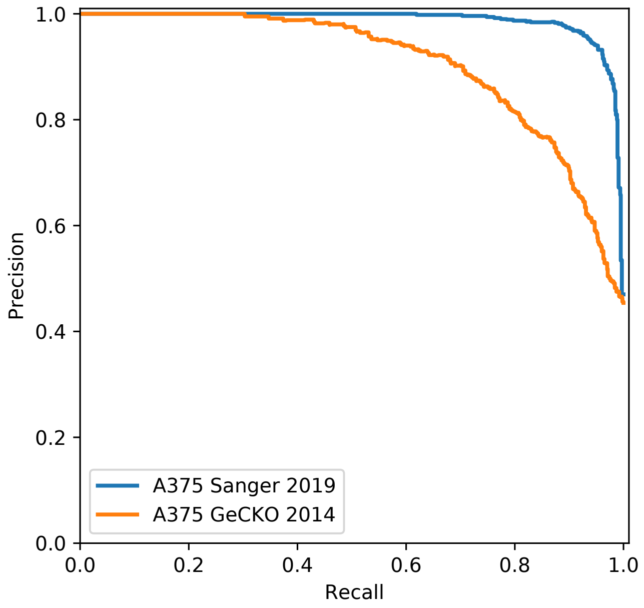

Figure S5. Recall-precision plot of gene essentiality screens in A375 cells.
Raw data was processed by BAGEL and precision/recall curves were generated using gold standard essential and nonessential genes from (35).
